# Exploration of New Drug Candidate Derived from Antioxidants of Korean Native Halophytes: Control of *Acinetobacter baumannii* with Antipathogenic Activity

**DOI:** 10.3390/antiox13111334

**Published:** 2024-10-31

**Authors:** Jihee Lee, Ho Sung Kim, Jeong Woo Park, Bohyun Yun, Woo Young Bang, Ki Hwan Moon, Youngwan Seo

**Affiliations:** 1 Division of Convergence on Marine Science, Korea Maritime & Ocean University, Busan 49112, Republic of Korea; nini_cagi@naver.com (J.L.); hosung7701@naver.com (H.S.K.); jwpark@g.kmou.ac.kr (J.W.P.); 2 Ocean Science and Technology School, Korea Maritime & Ocean University, Busan 49112, Republic of Korea; 3 Department of Convergence Interdisciplinary Education of Maritime & Ocean Contents, Korea Maritime & Ocean University, Busan 49112, Republic of Korea; 4 Division of Practical Research, Honam National Institute of Biological Resources, Mokpo-si 58762, Republic of Korea; boding3@hnibr.re.kr; 5 National Institute of Biological Resources, Environmental Research Complex, Incheon 22689, Republic of Korea; wybang@korea.kr

**Keywords:** *Acinetobacter baumannii*, anti-biofilm, anti-inflammation, antioxidant, antipathogenic, halophytes

## Abstract

The rise of antibiotic-resistant bacteria poses a significant challenge to the treatment of bacterial infections, necessitating the development of novel antibiotics or strategies to preserve the efficacy of existing ones. This study investigates the role of oxidative stress modulation in the pathogenicity of multidrug-resistant (MDR) bacterial strains, aiming to identify potential avenues for new drug design. Specifically, the anti-biofilm effects of crude extracts and fractions from seven halophyte species native to Jeju Island, South Korea, were evaluated against *Acinetobacter baumannii* ATCC 17978. Notably, the 85% aqueous methanol fraction of *Peucedanum japonicum* Thunb. (*Pj*) and the *n*-hexane fraction of *Lysimachia mauritiana* Lam. (*Lm*) demonstrated significant anti-biofilm activity. Further assessments revealed that these fractions also exhibited notable antioxidant and anti-inflammatory properties, with the *Pj* fraction showing a lifespan extension effect in the *Caenorhabditis elegans* model. These findings suggest that *Pj* and *Lm* hold promise as potential candidates for the development of new therapeutic agents targeting MDR bacteria.

## 1. Introduction

As we transition into the post-COVID-19 era, there is a heightened awareness of the risks posed by infectious diseases. The scientific community is increasingly focused on the timing, causative agents, and response strategies for the anticipated “Next Pandemic”. Many experts posit that the likelihood of a new pandemic emerging due to multidrug-resistant (MDR) bacteria is significantly high after COVID-19 [1,2]. They predict that the destructive power and impact of such a pandemic will be even greater than COVID-19, and the interval between its occurrences will become progressively shorter [3].

Antibiotics have played a crucial role in treating and preventing bacterial diseases. These antibiotics are produced in 100,000 tons annually, enabling the treatment of most bacterial infectious diseases [4]. However, bacteria have developed mechanisms to resist these antibiotics. These resistance mechanisms include mutations leading to alterations in target proteins, the enzymatic inactivation of antibiotics, the bypassing of the target pathway, and the use of efflux pumps to export the antibiotics [5]. These adaptations allow bacteria to survive in the presence of antibiotics. Moreover, the misuse and overuse of antibiotics has led to the emergence and spread of MDR bacteria, thereby increasing the selective pressure on antibiotics [6,7]. Recently, organizations such as the World Health Organization (WHO), the World Organisation for Animal Health (WOAH), the G20 Summit, and the Global Health Security Agenda (GHSA) have underscored the severity of the antibiotic resistance problem and called for national-level responses [8,9]. Reports from the WHO and Public Health England indicate that approximately 700,000 people die annually from MDR bacterial infections. This figure is projected to rise to 10 million per year by 2050, with an estimated economic loss exceeding $100 trillion USD annually [10]. To combat and treat MDR bacteria, research is actively focusing not only on the discovery of new antibiotics but also on the development of novel therapeutic techniques utilizing antipathogenic substances, antioxidants, host immunity enhancers, probiotics, antimicrobial peptides, and bacteriophages [11,12]. The significance of approaches employing new concepts, new methods, and new sources is becoming evident in the quest to discover these new bioactive substances.

*Acinetobacter baumannii* is a Gram-negative, aerobic, non-motile, and opportunistic pathogen. *A. baumannii* is one of the ESKAPE pathogens (*Enterococcus faecium*, *Staphylococcus aureus*, *Klebsiella pneumoniae*, *Acinetobacter baumannii*, *Pseudomonas aeruginosa*, and *Enterobacter* species), which primarily causes nosocomial infections by infecting the skin, respiratory tract, and digestive system. Recently, it has contributed to significant morbidity and mortality worldwide due to urinary tract infections. Therefore, antibiotic treatment is essential to treat infections caused by *A. baumannii* [13,14]. Unfortunately, previous studies reported that hospital-isolated *A. baumannii* have multidrug resistance, and some of the *A. baumannii* strains isolated from hospital have pandrug resistance [15]. Due to these issues, carbapenem-resistant *A. baumannii* has been considered priority 1 (critical) on the WHO list of priority pathogens for the research and development of new antibiotics [16]. This *A. baumannii* possesses various virulence factors such as outer membrane proteins (OMPs), protein secretion systems, lipopolysaccharides (LPSs), phospholipases, proteases, and iron (Fe)-chelating systems, and it forms biofilms, which contribute to pathogenicity by causing chronic infections and making them more difficult to treat [13,17].

Biofilm is recognized as a major virulence factor for various infectious bacteria, not only in *A. baumannii,* acting as a strategy for microorganisms to adapt and survive in hostile environments. Pathogenic bacteria forming biofilms are particularly difficult to eradicate and exhibit much greater antibiotic resistance, making them highly challenging to treat [18]. Major strategies to eliminate biofilms have included inhibiting bacterial attachment and colonization on surfaces, interfering with signal molecules that regulate biofilm development, and degrading the biofilm matrix. However, due to various limitations, the need for alternative approaches to effectively prevent biofilm formation has been emphasized [19,20,21]. Oxidative stress is one of the primary mechanisms prompting microorganisms to transition from a planktonic state to a biofilm-forming sessile state [22]. Therefore, chemicals targeting oxidative stress regulators, such as antioxidants, are considered potential treatments for biofilm-related infections.

Historically, mankind has utilized various herbs to treat diseases and wounds, and the accumulated knowledge of these herbs is currently used as a guide for developing many natural-source pharmaceuticals [23]. However, with the extension of the human lifespan and the emergence of new diseases, the need for new drug development has rapidly increased, while the quantity and diversity of terrestrial resources have gradually reached their limits due to prolonged research. To address this issue, attention has shifted to marine and the surrounding coastal biological resources [24,25]. Approximately 80% of life forms on Earth are found in the ocean, and research on marine species remains insufficient. Additionally, since marine organisms inhabit environments different from terrestrial ones, there is a high likelihood of discovering unique biochemical metabolites distinct from those of terrestrial organisms [26].

Halophytes are plants that grow in high-salinity soils such as coastal sand dunes or mudflats [27]. Unlike terrestrial plants, halophytes are known for their strong salt tolerance, achieved by expelling absorbed salt or maintaining cellular osmotic pressure. High-salinity environments induce significant oxidative stress in halophyte tissues, reducing the gas exchange in plants, decreasing the carbon dioxide supply to leaf tissues, and, ultimately, reducing photosynthetic electron transport, leading to the generation of various reactive oxygen species (ROS) [28]. To resist this salt-induced oxidative stress, halophytes have developed unique antioxidant mechanisms, and numerous antioxidants have been produced as self-defense substances. Globally, approximately 1560 species of halophytes have been identified, with 72 species confirmed in South Korea [29,30].

This study aims to verify the anti-biofilm effects of crude extracts and fractions obtained from various Korean native halophytes on the *A. baumannii* ATCC 17978 strain, and to investigate their antioxidant and anti-inflammatory properties. Additionally, we conducted in vitro cytotoxicity evaluations, and a lifespan extension experiment using the *Caenorhabditis elegans* model to confirm the potential for in vivo applications. Through this research, we aim to assess the impact of antioxidants and anti-inflammatory substances from halophytes on the pathogenicity of MDR bacteria and evaluate the potential for new drug development designs. The assessment of the antipathogenic (anti-biofilm) capabilities of antioxidant substances is consistent with the concept of drug repositioning for the management of MDR bacteria. Ultimately, this research aims to validate the potential of utilizing antioxidant substances derived from Korean native halophytes as novel therapeutics and to generate high added value from blue carbon biomass.

## 2. Materials and Methods

### 2.1. Chemicals

Methylene chloride, methanol, *n*-butanol, *n*-hexane, DMSO, and other solvents were purchased from Duksan (Ansan, Republic of Korea). Fetal bovine serum (FBS) was obtained from Gibco (Thermo Fisher Scientific, Waltham, MA, USA) and penicillin/streptomycin solution was purchased from Corning (Corning, NY, USA). RPMI 1640 medium, Dulbecco’s Modified Eagle’s Medium (DMEM) and phosphate-buffered saline (PBS) were obtained from Bylabs (Suwon, Republic of Korea). Modified Eagle Medium (MEM) was obtained from Gibco (Thermo Fisher Scientific, Waltham, MA, USA). 3-(4,5-dimethylthiazol-2-yl)-2,5-diphenyltetrazolium bromide reagent, 2′,7′-Dichlorodihydrofluorescein diacetate (DCFH-DA), monobromobimane (mBBr), and Lipopolysaccharide (LPS) were obtained from Sigma Chemical Co. (St. Louis, MO, USA). Luria–Bertani (LB) broth and Bacto Agar were obtained from BD biosciences (Franklin Lakes, NJ, USA). For *C. elegans* culture, Nematode Growth Medium (NGM) was obtained from Research Products International (Chicago, IL, USA). Streptomycin and nystatin were obtained from Sigma-Aldrich (St. Louis, MO, USA).

### 2.2. Extraction and Fractionation of Korean Native Halophytes

**Figure 1:**
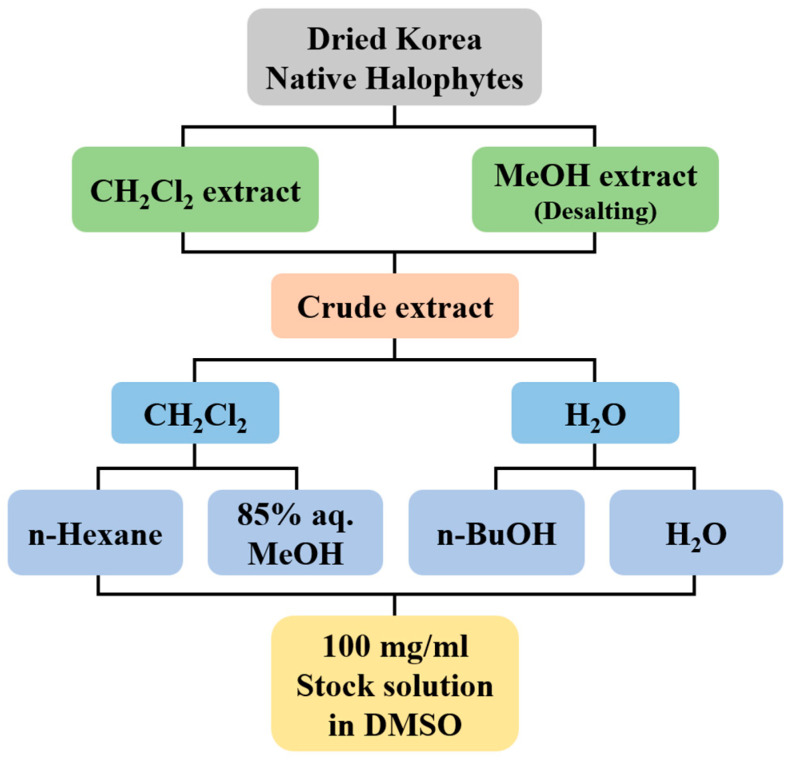
Flow chart for preparation of crude extract and its solvent fractions from Korean native halopytes.

The Korean native halophytes used in this study are listed in Table 1. Additionally, these halophytes were collected from Jeju Island, Republic of Korea. The collected halophytes were extracted twice with dichloromethane (CH_2_Cl_2_) and twice with methanol (MeOH) for 24 h, respectively. After desalting, combined crude extracts were fractionated with CH_2_Cl_2_ and water. The aqueous layers were further fractionated into *n*-butanol (*n*-BuOH) and water, and the organic layers were also fractionated into *n*-hexane (*n*-Hex) and 85% aqueous MeOH (85% aq. MeOH) to afford *n*-Hex, 85% aq. MeOH, *n*-BuOH, and water fractions. Each of the obtained fractions, including crude extract, was dried and concentrated using a rotary evaporator, then resuspended in DMSO at a concentration of 100 mg/mL. And the stock solutions were restored at 4 °C (Figure 1).

### 2.3. Bacterial Strains and Growth Conditions

*Acinetobacter baumannii* ATCC 17978 strain, and *Escherichia coli* OP50 strain were used in this study. Both strains were stored in 15% peptone–glycerol solution at −80 °C. The bacterial strains were routinely cultured in Luria–Bertani (LB) liquid medium or on LB agar plates at 37 °C. For liquid cultures, shaking was performed at 200 rpm. A cocktail containing 30 μg/mL of sulfamethoxazole (Smx) and 6 μg/mL of trimethoprim (Tmp), and DMSO (100 μg/mL) were used as control, when necessary [31].

### 2.4. Caenorhabditis elegans Strain and Growth Condition

The *C. elegans* N2 strain wild-type (*Caenorhabditis* Genetic Center) and AU37 strain (*glp-4*(*bn2*) I; *sek-1*(*km4*) X) were obtained from the *Caenorhabditis* Genetics Center (CGC) and used in these experiments. Cultivation of all worms used here was maintained at 20 °C on nematode growth medium (NGM) plates supplemented with streptomycin (100 μg/mL) and nystatin (50 units/mL), seeded with *E. coli* OP50. Synchronization of worms for these experiments was performed using standard protocols [32]. Briefly, worms were cultivated on NGM to obtain large numbers of gravid adult hermaphrodites, and eggs were isolated from these plates via bleach–NaOH lysis of adults. Eggs were rinsed 5 to 6 times in M9 buffer to remove the hypochlorite solution and allowed to hatch in 5 mL of M9 buffer. After overnight incubation, synchronized and starved L1 stage *C. elegans* were cultured in NGM plate seeded with *E. coli* OP50, and used for experiments.

### 2.5. Cell Culture

Human A549 lung adenocarcinoma cells, and RAW 264.7 macrophages used in the experiments were obtained from the Korean Cell Line Bank (KCLB). The A549 cell line was cultured in RPMI 1640 medium (Bylabs, Suwon, Republic of Korea) supplemented with 10% fetal bovine serum (FBS) and 1% penicillin/streptomycin, under a humidified atmosphere at 37 °C with 5% CO_2_.

Specifically, for the NO assay, the RAW 264.7 cells were cultured in Dulbecco’s Modified Eagle’s Medium (DMEM, Bylabs, Suwon, Republic of Korea) supplemented with 10% fetal bovine serum (FBS) and 1% penicillin/streptomycin under the same conditions. The cells were seeded in a 96-well plate and allowed to adhere 24 h prior to sample treatment.

### 2.6. Biofilm Formation Analysis

An overnight culture of *Acinetobacter baumannii* 17978 was inoculated at 1% into a polystyrene tube (500 μL of medium per tube), with the addition of halophyte extract or fraction. The culture was then incubated under aerobic conditions (37 °C, 200 rpm) for 24 h. Kinetics of bacterial cell growth was measured by using an Epoch2 microplate Spectrophotometer (BioTek, Winooski, VT, USA) at 600 nm wavelength.

Bacterial cell culture broths were discarded from the culture tubes. The cell culture tubes were washed five times with water and then stained with crystal violet (CV). Six hundred microliters of 0.1% CV (dissolved in water) were added. The culture tubes were incubated for 10 min at room temperature before removal of the CV staining solution, and then were washed five times with water. After washing, 600 μL of 10% acetic acid solution was added to dissolve the biofilm-bound CV by gently shaking the culture tubes. To quantify the biofilm formation ability, CV dissolved in acetic acid solution was dispensed onto sterile, clear, round-bottomed, 96-well, polystyrene plates (Corning Inc., Corning, NY, USA) (200 μL per well), and absorbance was measured at 550 nm. DMSO and Smx/Tmp cocktail were used as negative and positive control, respectively. All experiments were biological-triplicated and analyzed by one-way ANOVA. Error bars represent the standard deviation of three technical replicates.

### 2.7. MTT Cytotoxicity Assay

Cell viability in response to the samples was measured using the 3-(4,5-dimethylthiazol-2-yl)-2,5-diphenyltetrazolium bromide reagent (MTT assay). A549 cells were dispensed into 96-well plates at a density of 1 × 10^7^ cells/well and incubated for 24 h at 37 °C in a 5% CO_2_ incubator. Following incubation, the cells were exposed to various concentrations of the samples for 24 h. Phosphate-buffered saline (PBS, Bylabs, Suwon, Republic of Korea) was used as a control under the same conditions. After 24 h, 500 μg/mL of MTT reagent (Sigma, St. Louis, MO, USA) was added to each well, and the cells were incubated for an additional 4 h. The supernatant was then carefully removed, and the formazan crystals formed were dissolved in dimethyl sulfoxide (DMSO). Absorbance was measured at 540 nm using a Victor3 multilabel plate reader (PerkinElmer, San Diego, CA, USA). The experiments were performed in biological triplicate.

### 2.8. Measurement of Intracellular Reactive Oxygen Species (ROS)

To quantify the scavenging of intracellular free radicals, 2′,7′-Dichlorodihydrofluorescein diacetate (DCFH-DA, Sigma, St. Louis, MO, USA) was used. A549 cells were dispensed into black 96-well plates at a density of 1 × 10^7^ cells/well and incubated at 37 °C in a 5% CO_2_ incubator for 24 h. Subsequently, 20 μM DCFH-DA was added to each well and incubated for 20 min. After incubation, the cells were treated with samples at various concentrations and incubated under the same conditions for 1 h. The medium was then removed, and the cells were washed three times with PBS buffer. Subsequently, 500 μM H_2_O_2_ was added, and DCF fluorescence was measured at 30 min intervals from 0 to 120 min using a Victor3 multilabel plate reader with excitation at 485 nm and emission at 528 nm.

A blank (neither sample- nor H_2_O_2_-treated) and a negative control (without being sample-treated, but H_2_O_2_-treated) were used to evaluate reactive oxygen species (ROS) scavenging activity. All experiments were conducted in the dark room due to the light sensitivity of DCFH-DA and were performed in biological triplicate.

### 2.9. Measurement of Intracellular Reduced Glutathione (GSH)

To measure the content of accumulated intracellular glutathione (GSH), the thiol-reactive reagent monobromobimane (mBBr, Sigma, St. Louis, MO, USA ) was used. A549 cells were dispensed into black 96-well plates at a density of 1 × 10^7^ cells/well and incubated for 24 h at 37 °C in a 5% CO_2_ incubator. Each well was then treated with samples at various concentrations and incubated under the same conditions for 30 min. The medium was removed, and the cells were washed twice with PBS buffer. Subsequently, 40 μM mBBr was added and allowed to react under the same conditions for 60 min. Changes in GSH levels due to sample treatment were measured at an excitation wavelength of 360 nm and an emission wavelength of 465 nm using a microplate reader. PBS was used as a control under the same conditions. All experiments were conducted in the dark room due to the light sensitivity of mBBr and were performed in biological triplicate.

### 2.10. Determination of Nitric Oxide (NO) Production by RAW 264.7 Cells

To evaluate the scavenging activity on nitric oxide (NO), a type of ROS, NO assay was performed. RAW 264.7 macrophages were dispensed into 96-well plates at a density of 5 × 10^4^ cells/well and incubated for 24 h at 37 °C in a 5% CO_2_ incubator. Then, the medium was replaced with Modified Eagle Medium (MEM, Gibco, Waltham, MA, USA) containing 10% FBS, and the cells were treated with various concentrations of the samples and incubated for 1 h. Subsequently, to induce NO production, lipopolysaccharide (LPS, Sigma, St. Louis, MO, USA) was added at a concentration of 0.5 μg/mL (0.5 ppm) and the cells were incubated under the same conditions for 48 h. The NO produced by the LPS-stimulated cells was measured by reacting the culture medium with Griess reagent (Sigma, St. Louis, MO, USA) diluted in triple-distilled water at a ratio of 1:1, and the absorbance was measured at 570 nm. For the control groups, a blank without sample and LPS, and a negative control group without sample were used. The experiments were performed in biological triplicate.

### 2.11. C. elegans Life Span Analysis

The lifespan analysis of *C. elegans* was conducted using the N2 strain to assess the in vivo toxicity of a halophyte fraction. The *C. elegans* N2 strain was routinely maintained as previously described, seeded with *E. coli* OP50. Toxicity was measured according to previously described methods, with slight modifications [33]. Bleached *C. elegans* were cultured on NGM plates seeded with *E. coli* OP50. The cultured L1 stage *C. elegans* were harvested in 10 mL of M9 buffer, centrifuged at 2500 rpm for 2 min, washed three times with M9 buffer, and additionally washed once with 10 mL of S-complete. The worms were then resuspended in S-complete medium to a concentration of 80–100 worms/mL, and carbenicillin was added to a final concentration of 50 μg/mL along with Amphotericin B at 0.1 μg/mL. Then, 120 μL aliquot of this worm suspension was dispensed into each well of a 96-well plate and incubated until the worms reached the L4 stage. At the L4 stage, 30 μL of 0.6 mM FUDR was added to sterilization. One day later, to prevent starvation, 5 μL of 2 × 10^10^ CFU/mL *E. coli* OP50 was added once a week. Observations were conducted every day, and the number of live worms was counted based on their movement.

### 2.12. Bacterial Colonization Analysis in C. elegans Gut

The *C. elegans* strain AU37 (*glp-4*(*bn2*) I; *sek-1*(*km4*) X) was used in this experiment. This *C. elegans* strain was routinely maintained according to the method described in *2.3.*, and incubated at 25 °C for sterilization. To assess the reduced colonization ability of *A. baumannii* due to biofilm reduction, colonization of the *C. elegans* intestinal tract was performed according to previously described method, with slight modifications [34]. Briefly, L4/young-adult *C. elegans* was exposed for 2 days on an NGM plate supplemented with *Pj* 85% aq. MeOH or *Lm n*-Hex, 200 μg/mL concentration, respectively, and seeded with *A. baumannii* ATCC17978 as a food source. After 2 days, 10 worms were randomly selected, and washed twice using M9 buffer, and placed on brain-heart infusion agar containing kanamycin (100 μg/mL) and streptomycin (100 μg/mL) concentration. Additionally, *C. elegans* were exposed to gentamycin (25 μg/mL) for 5 min to remove all external bacteria, and washed three times using M9 buffer. Ten specimens were homogenized using the Power Masher II (Nippi, Tokyo, Japan), resuspended in 1 mL of PBS, and the bacterial count was determined via viable count. All experiments were technical-triplicated and analyzed by one-way ANOVA. Error bars represent the standard deviation of three technical replicates.

### 2.13. Statistical Analysis

All experimental data were statistically analyzed using GraphPad Prism 9.0 (GraphPad Software, San Diego, CA, USA). The specific statistical tests applied for each experiment are detailed in the corresponding figure legends. A *p*-value of ≤0.05 was considered statistically significant.

## 3. Results

### 3.1. Anti-Biofilm Activity Screening of Crude Extracts of Korean Native Halophytes

**Figure 2:**
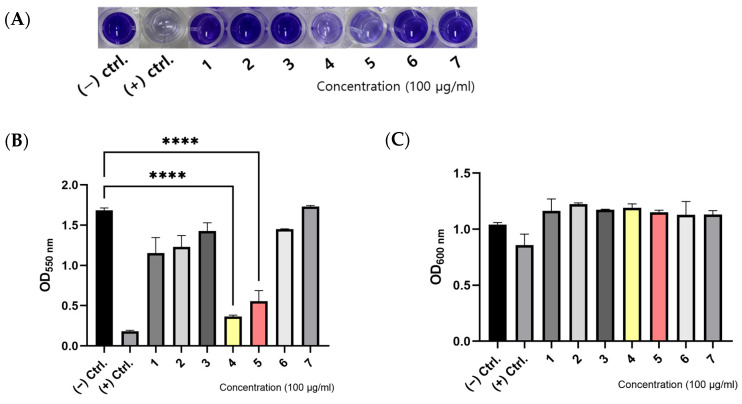
Effect of Korean native halopyte crude extracts with anti-biofilm activity on *A. baumannii* ATCC 17978: (**A**) representative crystal violet assay image, and (**B**) quantitative determination of biofilm formation of *A. baumannii* ATCC 17978 on polystyrene surface treated with seven different Korean native halopyte crude extracts; (**C**) kinetics of bacterial cell growth: 1. *Boehmeria pannosa* Nakai & Satake ex Oka; 2. *Carex pumila* Thunb.; 3. *Carex kobomugi* Ohwi; 4. *Peucedanum japonicum* Thunb.; 5. *Lysimachia mauritiana* Lam.; 6. *Asparagus cochinchinensis* Merr.; and 7. *Artemisia fukudo* Makino; values are the mean ± STDEV of the mean of three replicates per group. One-way ANOVA followed Dunnett’s test. **** *p* < 0.0001.

The impact of various samples on biofilm formation by *Acinetobacter baumannii* ATCC 17978 was quantitatively evaluated using crystal violet staining (Figure 2A,B). Crude extracts from seven Korean native halophytes, collected from Jeju Island, were prepared in order to assess their anti-biofilm activity. The species examined includued *Boehmeria pannosa* Nakai & Satake ex Oka, *Carex pumila* Thunb., *Carex kobomugi* Ohwi, *Peucedanum japonicum* Thunb., *Vitex rotundifolia*, *Lysimachia mauritiana* Lam., *Asparagus cochinchinensis* Merr., and *Artemisia fukudo* Makino, as shown in Table 1. Each crude extract was administered at a concentration of 100 μg/mL. Notably, the crude extracts from *Peucedanum japonicum* Thunb (*Pj*) and *Lysimachia mauritiana* Lam. (*Lm*) exhibited a significant inhibition of biofilm formation, reducing it by 78.2% and 67.0%, respectively. These inhibitory effects were comparable to those observed in the positive control group treated with an Smx/Tmp cocktail. In contrast, DMSO, employed as a negative control at an equivalent concentration, showed no impact on biofilm formation. Based on these preliminary findings, further experiments were focused on the two most promising samples, *Pj* and *Lm*, to elucidate their potential therapeutic applications as a new drug candidates.

Since biofilm formation, as measured by crystal violet staining, reflects both the biofilm structure and bacterial growth, it is essential that we ascertain whether the observed anti-biofilm effects are due to the inhibition of bacterial growth. To address this, the absorbance of bacterial suspensions cultured in polystyrene tubes was measured at 600 nm (Figure 2C). No significant inhibition of bacterial growth was observed for any of the samples tested. These results suggest that the crude extracts of *Pj* and *Lm*, which demonstrated significant anti-biofilm activity, exert their effects by specifically inhibiting biofilm formation without affecting the overall growth of *A. baumannii*.

### 3.2. Anti-Biofilm Activity of Different Fractions of Peucedanum japonicum Thunb. and Lysimachia mauritiana Lam.

**Figure 3:**
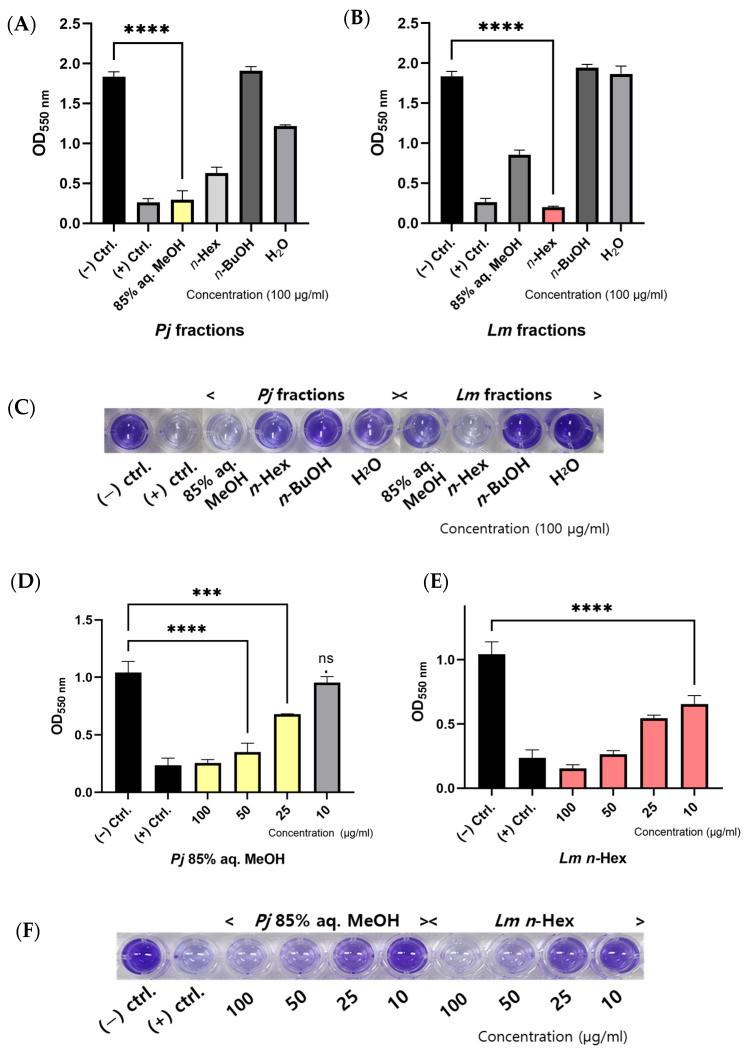
Anti-biofilm activity of *P. japonicum* Thunb. or *L. mauritiana* Lam. fractions againt *A. baumannii* ATCC 17978: (**A**,**B**) quantitative determination of biofilm formation, and (**C**) representative crystal violet assay image of *A. baumannii* ATCC 17978 on polystyrene surface with *P. japonicum* Thunb. or *L. mauritiana* Lam. fractions; concentration-dependent effect of *P. japonicum* Thunb. 85% aq. MeOH fraction or *L. mauritiana* Lam. *n*-Hex fraction on *A. baumannii* ATCC 17978 biofilm formation; (**D**,**E**) quantitative measurements of biofilm formation; and (**F**) representative crystal violet assay image of *A. baumannii* ATCC 17978 with each fractions. Values are the mean ± STDEV of the mean of three replicates per group. One-way ANOVA followed Dunnett’s test. *** *p* < 0.001, **** *p* < 0.0001.

To further investigate the *Pj* and *Lm* crude extracts, which had shown high anti-biofilm efficacy, the crude extracts were fractionated into 85% aqueous methanol (aq. MeOH), *n*-hexane (*n*-Hex), *n*-butanol (*n*-BuOH), and water fractions (Figure 3A–C). Each fraction was tested at a concentration of 100 μg/mL. The results revealed that both *Pj* and *Lm* samples significantly inhibited biofilm formation by more than 50% in most fractions, with the exception of the *n*-BuOH and water fractions. Notably, the 85% aq. MeOH fraction of *Pj* and *n*-Hex fraction of *Lm* exhibited the most potent anti-biofilm activity, reducing biofilm formation by 83.9% and 89.0%, respectively. These levels of inhibition were comparable to those observed in the positive control group treated with an Smx/Tmp cocktail.

We conducted an additonal crystal violet assay to evaluate the dose-dependent effects of the 85% aq. MeOH fraction of *Pj* and the *n*-Hex fraction of *Lm*, both of which demonstrated significant anti-biofilm activity in the previous experiment (Figure 3D–F). Each fraction was tested at concentrations of 10, 25, 50, and 100 μg/mL. The results revealed a clear dose-dependent inhibition of biofilm formation by both fractions. Specifically, the 85% aq. MeOH fraction of *Pj* reduced biofilm formation by 8.6%, 34.6%, 66.4%, and 75.4% at 10, 25, 50, and 100 μg/mL, respectively. Similarly, the *n*-Hex fraction of *Lm* decreased biofilm formation by 37.0%, 47.8%, 74.6%, and 85.1% at the same concentrations.

### 3.3. In Vitro Cytotoxicity Assay Against Human Lung Epithelial Cell A549

**Figure 4:**
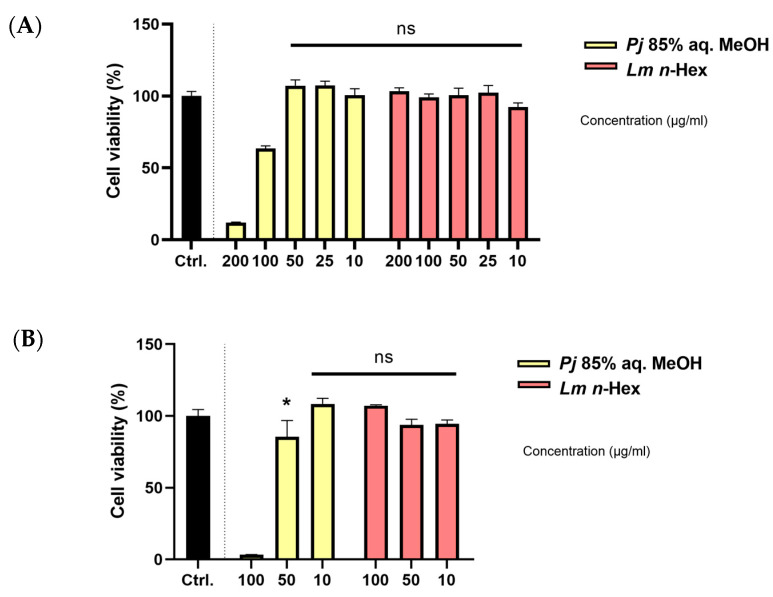
Percentage of lethality in (**A**) A549 cells or (**B**) RAW 264.7 cells after exposure to *P. japonicum* Thunb. 85% aq. MeOH fraction or *L. mauritiana* Lam. *n*-Hex fraction. Values are expressed as percentages compared to the control group and are the mean ± STDEV of the mean of three replicates per group. One-way ANOVA followed Dunnett’s test. * *p* < 0.05.

The cytotoxic effects of the 85% aq. MeOH fraction of *Pj* and the *n*-Hex fraction of *Lm* on A549 cells were evaluated using the MTT assay (Figure 4A). A549 cells were treated with each fraction at concentrations of 10, 25, 50, 100, and 200 μg/mL for 24 h. The results indicated that the 85% aq. MeOH fraction of *Pj* maintained over 80% cell viability at concentrations of up to 50 μg/mL. In contrast, the *n*-Hex fraction of *Lm* maintained an over 80% cell viability at concentrations of up to 200 μg/mL. Based on these results, concentrations of 10, 25, 50, 100, and 200 μg/mL were selected for subsequent experiments, except for the nitric oxide (NO) assay.

The effect of these fractions on the viability of RAW 264.7 macrophages, used in the NO assay, was also assessed using the same MTT assay method (Figure 4B). The experiment was conducted at concentrations of 10, 50, and 100 μg/mL. Similar to the observations in A549 cells, the 85% aq. MeOH fraction of *Pj* maintained an over 80% cell viability at concentrations of up to 50 μg/mL, while the *n*-Hex fraction of *Lm* maintained an over 80% cell viability at concentrations of up to 100 μg/mL. Consequently, concentrations of 10, 50, and 100 μg/mL were selected for use in the NO assay.

### 3.4. Antioxidant Responses Through Intracellular Reactive Oxygen Species (ROS) and Gluthathione (GSH) Levels

**Figure 5:**
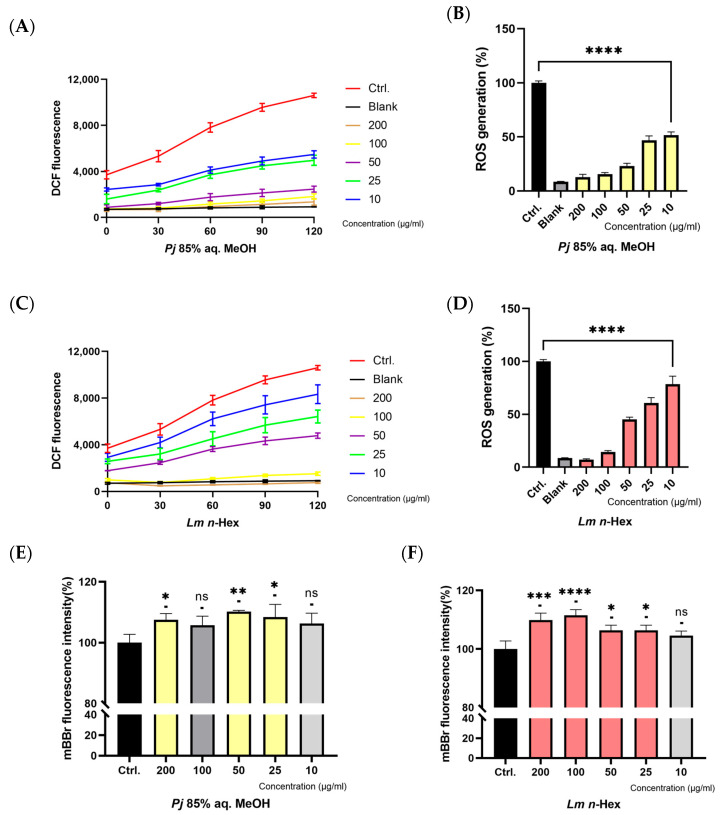
Concentration-dependent effect of *P. japonicum* Thunb. 85% aq. MeOH fraction or *L. mauritiana* Lam. *n*-Hex fraction on intracelluar ROS production and enhanced GSH levels; (**A**,**C**) intracellular ROS formation in A549 cells treated with each fractions at different concentrations; and (**B**,**D**) ROS scavenging activity of each fractions at different concentrations after 120 min. Values are expressed as percentages compared to the control group and are the mean ± STDEV of the mean of three replicates per group. One-way ANOVA followed Dunnett’s test. **** *p* < 0.0001; (**E**,**F**) Intracellular GSH level in A549 cells treated with each fractions at different concentrations. Values are expressed as percentages compared to the control group and are the mean ± STDEV of the mean of three replicates per group. One-way ANOVA followed Dunnett’s test. * *p* < 0.05, ** *p* < 0.01, *** *p* < 0.001, **** *p* < 0.0001.

Intracellular reactive oxygen species (ROS) levels were assessed by measuring DCF fluorescence using DCFH-DA, which emits fluorescence upon reacting with ROS (Figure 5A,B). The samples were treated at concentrations of 10, 25, 50, 100, and 200 μg/mL. A blank group and a control group were included to evaluate the ROS scavenging activity. The blank group was treated with only PBS, without the sample or H_2_O_2_, and the control group was treated with PBS in place of the sample, while H_2_O_2_ was treated in the same amounts as with the other groups. DCF fluorescence values were measured at 30 min intervals from 0 to 120 min. The negative control group exhibited a rapid increase in DCF fluorescence values over time, indicating substantial ROS formation, while the blank group showed minimal changes throughout the measurement period. Both experimental groups demonstrated a dose-dependent inhibition of ROS formation. After two hours, the 85% aq. MeOH fraction of *Pj* and the *n*-Hex fraction of *Lm* exhibited over 50% ROS scavenging activity at concentrations of 25 μg/mL and 50 μg/mL or higher, respectively, compared to the control group.

Intracellular glutathione (GSH) levels were quantified using monobromobimane (mBBr), a non-fluorescent compound that becomes fluorescent upon binding with GSH. GSH plays a critical role in scavenging intracellular ROS (Figure 5C,D). As a negative control, the control group was treated with PBS in place of the sample, while mBBr was treated in the same amounts as with the other groups. Compared to the control group, the results demonstrated that hte treatment with the 85% aq. MeOH fraction of *Pj* led to an increase in GSH levels by approximately 6.3%, 8.4%, 10.2%, 5.7%, and 7.5% at concentrations of 10, 25, 50, 100, and 200 μg/mL, respectively. Similarly, the treatment with the *n*-Hex fraction of *Lm* resulted in GSH level increases of approximately 4.5%, 6.3%, 6.3%, 11.5%, and 9.9% at the same respective concentrations. These results indicate that both fractions enhanced GSH levels at all tested concentrations compared to the negative control, with particularly significant increases of over 10% observed at 50 μg/mL of the 85% aq. MeOH fraction of *Pj* and 100 μg/mL of the *n*-Hex fraction of *Lm*.

### 3.5. Anti-Inflammatory Effect in Lipopolysaccharide (LPS)-Induced Macrophage RAW 264.7 Cells

**Figure 6:**
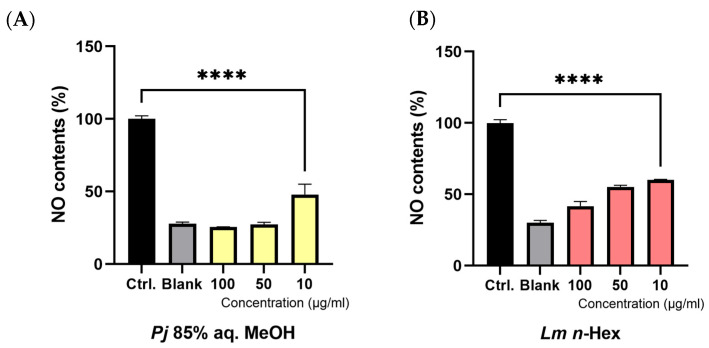
Concentration-dependent effect of (**A**) *P. japonicum* Thunb. 85% aq. MeOH fraction or (**B**) *L. mauritiana* Lam. *n*-Hex fraction on nitric oxide (NO) production by LPS-stimulated RAW 264.7 macrophage cells. Values are expressed as percentages compared to the control group and are the mean ± STDEV of the mean of three replicates per group. One-way ANOVA followed Dunnett’s test. **** *p* < 0.0001.

The anti-inflammatory effects of the 85% aq. MeOH fraction of *Pj* and the *n*-Hex fraction of *Lm* were evaluated by assessing the inhibition of NO production, a key inflammatory mediator (Figure 6A,B). The samples were tested at concentrations of 10, 50, and 100 μg/mL. As a negative control, the control group was treated with PBS in place of the sample, while lipopolysaccharide (LPS) was treated in the same amounts as with the other groups. And the blank group was treated with only PBS, without the sample or LPS. Compared to the control group, the 85% aq. MeOH fraction of *Pj* exhibited NO inhibition rates of approximately 52.1%, 72.7%, and 74.5% at 10, 50, and 100 μg/mL, respectively. Similarly, the *n*-Hex fraction of *Lm* showed NO inhibition rates of approximately 39.7%, 44.4%, and 58.3% at the same respective concentrations. Notably, the 85% aq. MeOH fraction of *Pj* demonstrated a particularly strong inhibitory effect at 50 and 100 μg/mL, with results closely resembling those of the blank group, which was not stimulated by LPS.

### 3.6. In Vivo Toxicity and Life Span Test Using Caenorhabditis elegans Model

**Figure 7:**
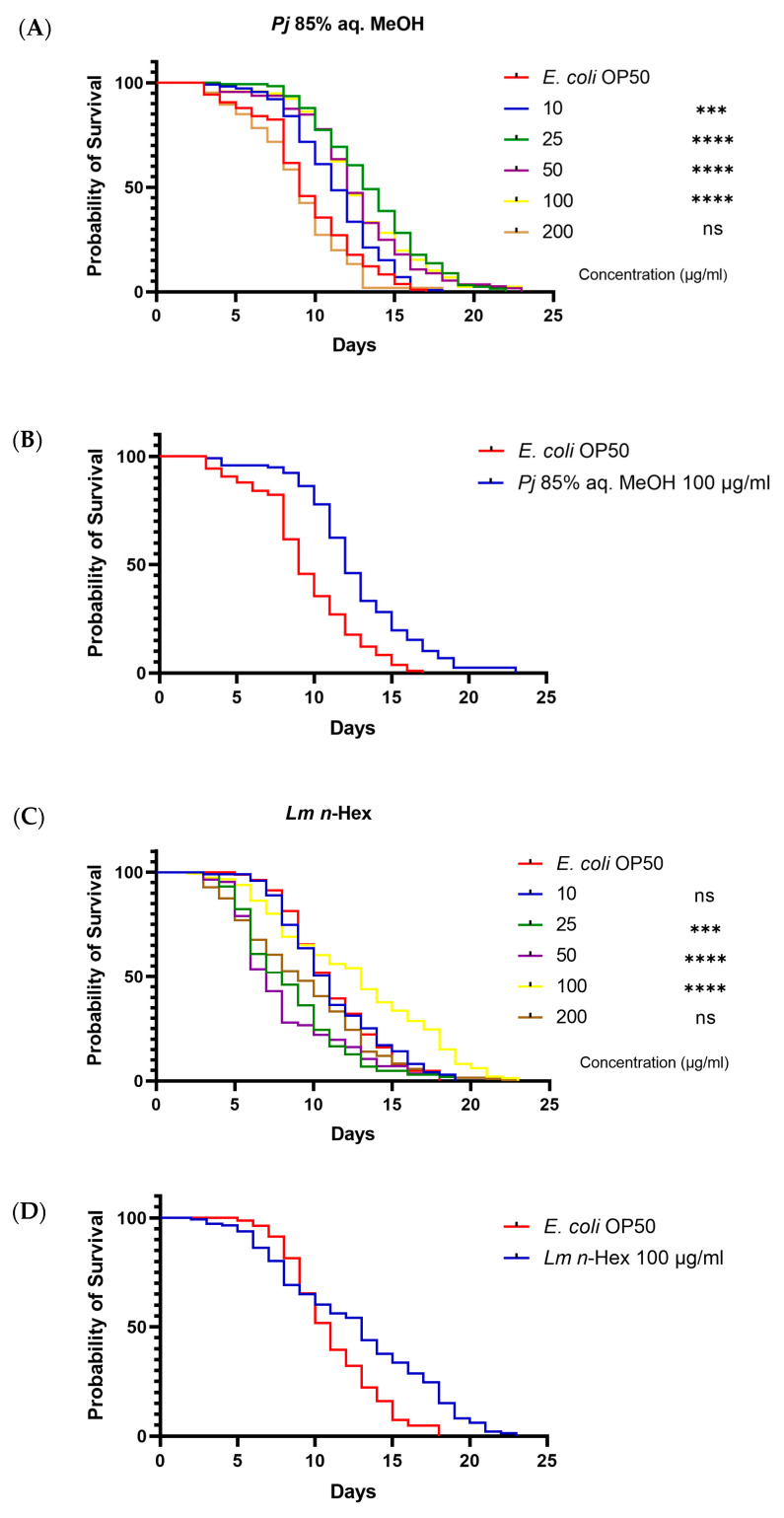
*C. elegans* survival analysis with *P. japonicum* Thunb. 85% aq. MeOH fraction or *L. mauritiana* Lam. *n*-Hex fraction: (**A**) concentration-gradient survival curve of *P. japonicum* Thunb. 85% aq. MeOH fraction; (**B**) survival curve of *P. japonicum* Thunb. 85% aq. MeOH fraction with 100 μg/mL of concentration; (**C**) concentration-gradient survival curve of *L. mauritiana* Lam. *n*-Hex fraction; and (**D**) survival curve of *L. mauritiana* Lam. *n*-Hex fraction with 100 μg/mL of concentration. A log-rank (Mantel–Cox) test was used to compare the mortality of all concentration relative to *E. coli* OP50 and significance is indicated. *** *p* < 0.001, **** *p* < 0.0001.

To evaluate the in vivo toxicity of the 85% aq. MeOH fraction of *Pj*, and the *n*-Hex fraction of *Lm*, a lifespan assay was conducted using the *C. elegans* N2 strain model. The fractions were tested at concentrations of 10, 25, 50, 100, and 200 μg/mL. For the 85% aq. MeOH fraction of *Pj*, an increase in the lifespan of *C. elegans* was observed at concentrations of 10, 25, 50, and 100 μg/mL. At 200 μg/mL, the lifespan was similar to that of the *E. coli* OP50-fed negative control (Figure 7A,B). Similary, the *n*-Hex fraction of *Lm* significantly increased the lifespan of *C. elegans* at concentrations of 25, 50, and 100 μg/mL. At 10 and 200 μg/mL, however, the lifespan decreased to levels comparable to the negative control (Figure 7C,D). These results suggest that both fractions have the potential to extend the lifespan of *C. elegans*, but a concentration of 200 μg/mL in both fractions may induce cytotoxic effects, as evidenced by the reduced lifespan observed at this concentration.

### 3.7. In Vivo Colonization Ability Test Using Caenorhabditis elegans Model

**Figure 8:**
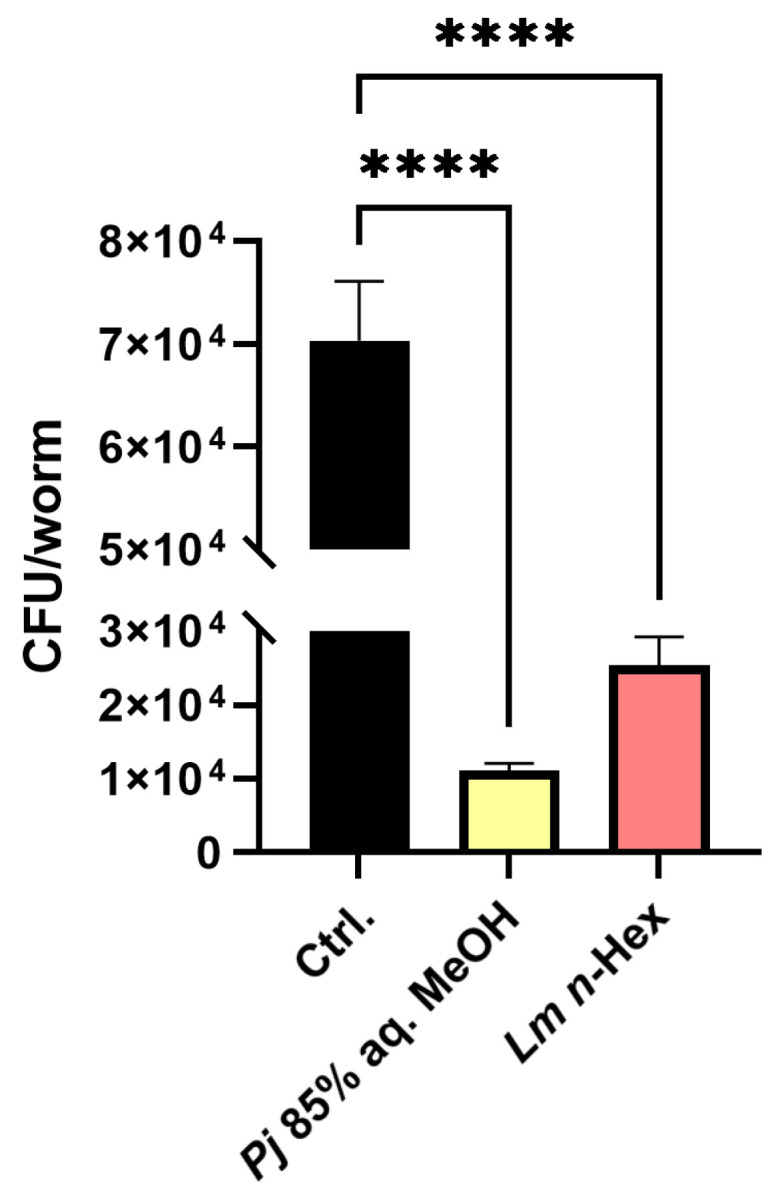
Colonization of *A. baumannii* in *C. elegans* gut treated with *P. japonicum* Thunb. 85% aq. MeOH fraction or *L. mauritiana* Lam. *n*-Hex fraction. Colony-forming units (CFUs) of *A. baumannii* treated with 200 μg/mL of each fraction in the worm gut. All experiments were technical-triplicated and analyzed by one-way ANOVA. Error bars represent the standard deviation of three technical replicates. **** *p* < 0.0001.

To confirm the decrease in adhesion ability due to the reduction in biofilm formation, the number of bacteria colonizing the intestines was measured using viable counts (Figure 8). The *C. elegans* AU37 strain (*glp-4*(*bn2*) I; *sek-1*(*km4*) X) was employed as an in vivo intesinal model. The worms were cultured on NGM plates supplemented with the 85% aq. MeOH fraction of *Pj*, the *n*-Hex fraction of *Lm*, and DMSO as a negative control. The results indicated that the number of intestinal *A. baumannii* 17978 bacteria in *C. elegans* fed on plates supplemented with the 85% aq. MeOH fraction of *Pj* was reduced by 84.19% compared to the DMSO negative control. In the case of supplementation with the *n*-Hex fraction of *Lm*, the intestianl *A. baumannii* 17978 bacterial load was reduced by 63.98% compared to the negative control.

## 4. Discussion

Antibiotic resistance has emerged as a critical public health challenge, significantly increasing morbidity and mortality rates worldwide [35]. The escalating prevalence of multidrug-resistant (MDR) pathogens underscores the urgent need for novel strategies to combat these infections, particularly as conventional antibiotics lose their efficacy. *Acinetobacter baumannii*, which is classified in the WHO priority pathogen critical group, is considered a bacterium in urgent need of new antibiotic development. This study explored the potential of natural substances derived from halophytes as alternative therapeutic agents to address this challenge without contributing to the further emergence of antibiotic-resistant strains.

Our research focused on the anti-biofilm and antioxidant properties of crude extracts and fractions from halophytes native to Jeju Island, South Korea. Among the tested species, *Peucedanum japonicum* Thunb. (*Pj*) and *Lysimachia mauritiana* Lam. (*Lm*) demonstrated significant anti-biofilm activity against *Acinetobacter baumannii*, an MDR pathogen with a high resistance rate. Notably, the 85% aq. MeOH fraction of *Pj* and the *n*-Hex fraction of *Lm* exhibited potent activity, comparable to that of the antibiotic cocktail used as a positive control [31]. Importantly, these fractions inhibited biofilm formation without affecting bacterial growth, suggesting that they selectively target biofilm-related mechanisms rather than exerting bactericidal effects.

Moreover, the study demonstrated that these fractions possess robust antioxidant properties, as evidenced by their ability to scavenge reactive oxygen species (ROS) in A549 cells, a human lung adenocarcinoma cell line. The data suggest that these fractions contain potent antioxidants capable of mitigating oxidative stress, a key factor in cellular senescence and various chronic diseases [36,37]. Additionally, the fractions effectively increased glutathione (GSH) levels, with the *n*-Hex fraction of *Lm* displaying low cytotoxicity, thereby supporting its potential therapeutic application [38].

The study also explored the anti-inflammatory potential of these fractions by assessing their impact on nitric oxide (NO) production. Both fractions exhibited significant inhibitory effects on NO synthesis, with increasing concentrations correlating with enhanced anti-inflammatory activity. This suggests that the antioxidant activity of these fractions may contribute to their ability to modulate inflammatory responses, a critical factor in the pathogenesis of chronic infections and other inflammatory diseases.

Using *Caenorhabditis elegans* as a model organism, the toxicity of the fraction was assessed. All concentrations of each fraction did not significantly reduce the life-span of *C. elegans*. These results indicate that the 85% aq. MeOH fraction of *Pj* and the *n*-Hex fraction of *Lm* exhibit little to no toxicity towards *C. elegans.* Moreover, the study further confirmed the potential anti-aging effects of the fractions, demonstrating a significant extension of the lifespan at various concentrations. This effect is likely attributable to the antioxidant and anti-inflammatory properties of the fractions, although further research is required to elucidate the precise mechanisms underlying this longevity [39]. Importantly, the reduction in biofilm formation by these fractions was also associated with a decrease in bacterial colonization within the intestine of *C. elegans*. This finding suggests that targeting biofilm formation could be an effective strategy for reducing bacterial pathogenicity, particularly in clinical settings where MDR strains pose a significant challenge [40,41,42].

Oxidative stress can promote bacterial stimulation, thereby enhancing biofilm formation, while inflammation occurs in biofilm-infected tissues, leading to damage at the infection site [20,43]. Thus, if these fractions are applied as candidates for controlling MDR pathogens, they could reduce the biofilm-associated pathogenicity while simultaneously alleviating oxidative stress and inflammatory responses at the infection site. This dual action may aid tissue recovery and reduce the immunological burden on the host, offering an integrated approach to infection suppression.

In conclusion, this study identifies the 85% aq. MeOH fraction of *Pj* and the *n*-Hex fraction of *Lm* as promising candidates for developing new strategies to control MDR pathogens. Their ability to inhibit biofilm formation, coupled with their antioxidant and anti-inflammatory activities, suggests that these natural substances could be integrated into existing treatment regimens to enhance the efficacy of conventional antibiotics. Future research should focus on isolating and characterizing the specific bioactive compounds responsible for these effects, as well as exploring their potential synergy with current antimicrobial therapies. Such efforts could pave the way for innovative approaches to managing bacterial infections in clinical settings, ultimately contributing to the global fight against antibiotic resistance.

## Figures and Tables

**Table 1:** Korean native halophytes used in this study.

No.	Sample Name	Sample Scientific Name
1	Seashore falsenettle	*Boehmeria pannosa* Nakai & Satake ex Oka
2	Dwarf sand sedge	* Carex pumila * Thunb.
3	Asian sand sedge	* Carex kobomugi * Ohwi
4	Coastal hogfennel	* Peucedanum japonicum * Thunb.
5	Spoon-leaf yellow loosestrife	* Lysimachia mauritiana * Lam.
6	Lucid asparagus	* Asparagus cochinchinensis * Merr.
7	Asian coastal wormwood	* Artemisia fukudo * Makino

## Data Availability

The data presented in this study are available upon request from the corresponding author.

## References

[1] Salazar C.B., Spencer P., Mohamad K., Jabeen A., Abdulmonem W.A., Fernandez N. (2022). Future pandemics might be caused by bacteria and not viruses: Recent advances in medical preventive practice. Int. J. Health Sci..

[2] Amodio E., Pizzo S., Vella G., De Francisci V., Distefano S.A., Giambelluca E., Graceffa D., Verso M.G., Piro E., Giuffre M. (2024). Increase of multidrug-resistant bacteria after the COVID-19 pandemic in a major teaching Hospital in Sicily (2018–2021). Int. J. Antimicrob. Agents.

[3] Ventola C.L. (2015). The antibiotic resistance crisis: Part 1: Causes and threats. Pharm. Ther..

[4] de Lima Procópio R.E., da Silva I.R., Martins M.K., de Azevedo J.L., de Araújo J.M. (2012). Antibiotics produced by Streptomyces. Braz. J. Infect. Dis..

[5] Nikaido H. (2009). Multidrug resistance in bacteria. Annu. Rev. Biochem..

[6] Malik B., Bhattacharyya S. (2019). Antibiotic drug-resistance as a complex system driven by socio-economic growth and antibiotic misuse. Sci. Rep..

[7] Llor C., Bjerrum L. (2014). Antimicrobial resistance: Risk associated with antibiotic overuse and initiatives to reduce the problem. Ther. Adv. Drug Saf..

[8] FAO, UNEP, WHO, WOAH (2022). One Health Joint Plan of Action (2022–2026). Working Together for the Health of Humans, Animals, Plants and the Environment.

[9] Shevade A., Naik S. (2023). Mitigation of Antimicrobial Resistance (AMR) in G20. Indian Public Policy Rev..

[10] O’Neill J. (2014). Antimicrobial Resistance: Tackling a Crisis for the Health and Wealth of Nations.

[11] Qadri H., Shah A.H., Mir M. (2021). Novel Strategies to Combat the Emerging Drug Resistance in Human Pathogenic Microbes. Curr. Drug Targets.

[12] Newman A.M., Arshad M. (2020). The Role of Probiotics, Prebiotics and Synbiotics in Combating Multidrug-Resistant Organisms. Clin. Ther..

[13] Howard A., O’Donoghue M., Feeney A., Sleator R.D. (2012). *Acinetobacter baumannii*: An emerging opportunistic pathogen. Virulence.

[14] Kyriakidis I., Vasileiou E., Pana Z.D., Tragiannidis A. (2021). *Acinetobacter baumannii* Antibiotic Resistance Mechanisms. Pathogens.

[15] Dijkshoorn L., Nemec A., Seifert H. (2007). An increasing threat in hospitals: Multidrug-resistant *Acinetobacter baumannii*. Nat. Rev. Microbiol..

[16] World Health Organization (2024). WHO Bacterial Priority Pathogens List, 2024: Bacterial Pathogens of Public Health Importance to Guide Research, Development and Strategies to Prevent and Control Antimicrobial Resistance.

[17] Shadan A., Pathak A., Ma Y., Pathania R., Singh R.P. (2023). Deciphering the virulence factors, regulation, and immune response to *Acinetobacter baumannii* infection. Front. Cell Infect. Microbiol..

[18] Stewart P.S., Costerton J.W. (2001). Antibiotic resistance of bacteria in biofilms. Lancet.

[19] Chen M., Yu Q., Sun H. (2013). Novel strategies for the prevention and treatment of biofilm related infections. Int. J. Mol. Sci..

[20] Kamaruzzaman N.F., Kendall S., Good L. (2017). Targeting the hard to reach: Challenges and novel strategies in the treatment of intracellular bacterial infections. Br. J. Pharmacol..

[21] Wu H., Moser C., Wang H.Z., Hoiby N., Song Z.J. (2015). Strategies for combating bacterial biofilm infections. Int. J. Oral Sci..

[22] Ong K.S., Mawang C.I., Daniel-Jambun D., Lim Y.Y., Lee S.M. (2018). Current anti-biofilm strategies and potential of antioxidants in biofilm control. Expert Rev. Anti Infect. Ther..

[23] Bremner M.R. (2011). Herbal Medicine: Biomolecular and Clinical Aspects.

[24] Montaser R., Luesch H. (2011). Marine natural products: A new wave of drugs?. Future Med. Chem..

[25] Papon N., Copp B.R., Courdavault V. (2022). Marine drugs: Biology, pipelines, current and future prospects for production. Biotechnol. Adv..

[26] Kim J., Choe S., Choe K., Lim S., Chai S. (2007). Funtional Components of Holophyte -Antioxidant substances in *Salicornia herbacea* L.. J. Fish. Mar. Sci. Educ..

[27] Rozema J., Bijwaard P., Prast G., Broekman R. (1985). Ecophysiological adaptations of coastal halophytes from foredunes and salt marshes. Vegetatio.

[28] Soundararajan P., Manivannan A., Jeong B.R., Gul B., Böer B., Khan M.A., Clüsener-Godt M., Hameed A. (2019). Different Antioxidant Defense Systems in Halophytes and Glycophytes to Overcome Salinity Stress. Sabkha Ecosystems: Volume VI: Asia/Pacific.

[29] Lee M., Kim S., Jung H. (2019). Distribution Patterns of Halophytes in the Coastal Area in Korea. Sea J. Korean Soc. Oceanogr..

[30] Shim H.B., Cho W.B., Choi B.H. (2009). Distribution of halophytes in coastal salt marsh and on sand dunes in Korea. Korean J. Plant Taxon..

[31] Moon K.H., Weber B.S., Feldman M.F. (2017). Subinhibitory Concentrations of Trimethoprim and Sulfamethoxazole Prevent Biofilm Formation by *Acinetobacter baumannii* through Inhibition of Csu Pilus Expression. Antimicrob. Agents Chemother..

[32] Stiernagle T. (2006). Maintenance of C. elegans.

[33] Solis G.M., Petrascheck M. (2011). Measuring Caenorhabditis elegans life span in 96 well microtiter plates. J. Vis. Exp..

[34] Park M.R., Yun H.S., Son S.J., Oh S., Kim Y. (2014). Short communication: Development of a direct in vivo screening model to identify potential probiotic bacteria using Caenorhabditis elegans. J. Dairy Sci..

[35] Salam M.A., Al-Amin M.Y., Salam M.T., Pawar J.S., Akhter N., Rabaan A.A., Alqumber M.A.A. (2023). Antimicrobial Resistance: A Growing Serious Threat for Global Public Health. Healthcare.

[36] Giorgio M., Trinei M., Migliaccio E., Pelicci P.G. (2007). Hydrogen peroxide: A metabolic by-product or a common mediator of ageing signals?. Nat. Rev. Mol. Cell Biol..

[37] Firsanov D.V., Solovjeva L.V., Svetlova M.P. (2011). H2AX phosphorylation at the sites of DNA double-strand breaks in cultivated mammalian cells and tissues. Clin. Epigenetics.

[38] Di Giacomo C., Malfa G.A., Tomasello B., Bianchi S., Acquaviva R. (2023). Natural Compounds and Glutathione: Beyond Mere Antioxidants. Antioxidants.

[39] Martel J., Wu C.Y., Peng H.H., Ko Y.F., Yang H.C., Young J.D., Ojcius D.M. (2020). Plant and fungal products that extend lifespan in Caenorhabditis elegans. Microb. Cell.

[40] Mishra R., Panda A.K., De Mandal S., Shakeel M., Bisht S.S., Khan J. (2020). Natural Anti-biofilm Agents: Strategies to Control Biofilm-Forming Pathogens. Front. Microbiol..

[41] Usman M., Yang H., Wang J., Tang J., Zhang L., Wang L. (2024). Formation, Regulation, and Eradication of Bacterial Biofilm in Human Infection.

[42] Ahumada-Santos Y.P., López-Angulo G., Pinto-González R.M., Clemente-Soto A.F., López-Valenzuela J.A., Delgado-Vargas F. (2024). Antibiofilm, cellular antioxidant, anti-inflammatory, immunomodulatory, cytotoxic, and antimutagenic activities of soluble melanins from *Randia echinocarpa* fruit. Adv. Tradit. Med..

[43] Yin S., Jiang B., Huang G., Gong Y., You B., Yang Z., Chen Y., Chen J., Yuan Z., Li M. (2017). Burn Serum Increases Staphylococcus aureus Biofilm Formation via Oxidative Stress. Front. Microbiol..

